# Oral Intake of EPA:DHA 6:1 by Middle-Aged Rats for One Week Improves Age-Related Endothelial Dysfunction in Both the Femoral Artery and Vein: Role of Cyclooxygenases

**DOI:** 10.3390/ijms21030920

**Published:** 2020-01-30

**Authors:** Sébastien Gaertner, Cyril Auger, Muhammad A. Farooq, Brigitte Pollet, Sonia Khemais-Benkhiat, Zahid R. Niazi, Sophie Schrevens, Sin-Hee Park, Florence Toti, Dominique Stephan, Valérie B. Schini-Kerth

**Affiliations:** 1 INSERM (French National Institute of Health and Medical Research), UMR 1260, Regenerativ.e Nanomedicine (RNM), FMTS, 67000 Strasbourg, France; 2 Service d’Hypertension et Maladies Vasculaires, Hôpitaux Universitaires de Strasbourg, 67000 Strasbourg, France; 3 UMR 7021, Laboratoire Bioimagerie et Pathologies, Faculté de Pharmacie, 67401 Illkirch, France

**Keywords:** endothelial dysfunction, aging, omega-3 polyunsaturated fatty acids, cyclooxygenases, venous thrombosis

## Abstract

In humans, aging is associated with endothelial dysfunction and an increased risk of venous thromboembolism. Although intake of eicosapentaenoic acid (EPA) and docosahexaenoic acid (DHA) at a ratio of 6:1 by old rats improved the endothelial dysfunction in arteries, the impact on veins remains unclear. Eight-month-old male Wistar rats were either untreated or orally administered corn oil, EPA:DHA 1:1, or EPA:DHA 6:1 (500 mg/kg/d) for seven days. Vascular reactivity was studied by myography. In middle-aged femoral artery rings, acetylcholine caused a partial relaxation at low concentrations and a contractile response at high concentrations, whereas in the old femoral vein only a partial relaxation was observed. The EPA:DHA 6:1 treatment blunted the contractile response to acetylcholine in the middle-aged femoral artery and both EPA:DHA 6:1 and 1:1 increased the relaxation to acetylcholine in the old femoral vein. No such effects were observed with corn oil. Both the non-selective cyclooxygenase inhibitor indomethacin and the COX-1 inhibitor SC-560 increased the relaxation to acetylcholine in the middle-aged femoral artery whereas the COX-2 inhibitor NS-398 increased that in the middle-aged femoral vein. In conclusion, our results indicate that aging is associated with an endothelial dysfunction in the femoral artery and vein, which can be improved by EPA:DHA 6:1 treatment—most likely via a cyclooxygenase-dependent mechanism.

## 1. Introduction

Venous thromboembolism (VTE), which includes deep vein thrombosis and pulmonary embolism, is a major cause of cardiovascular mortality [1,2]. The incidence of VTE is positively correlated with age, independent of the gender or ethnicity. Nevertheless, the identification of other risk factors for venous thrombosis remains a major issue in the prevention of VTE and its recurrence, and they are key determinants for the treatment intensity and duration [3]. The REMOTEV registry study recently indicated that the risk of unprovoked VTE, the severity of pulmonary embolism, and the risk of VTE recurrence are associated with cardiovascular risk factors, especially age and diabetes [4]. Nonetheless, up to 50% of VTEs remain unprovoked and thus lead to long-term anticoagulation therapy which exposes patients to an increased hemorrhagic risk [5,6,7,8]. However, almost one-third of patients are exposed to VTE recurrence within 10 years in spite of the long-term anticoagulation therapy [9]. In addition, studies report that VTE prevention is less effective in elderly patients, in the presence or absence of thromboprophylaxis, suggesting an age-dependent and anticoagulant-independent risk for VTE [10]. Thus, as VTE and bleeding risk increase with age, new therapeutic approaches for the prevention of VTE are needed [11].

Aging is associated with deleterious effects on the vascular system, at least in part following the overexpression of cyclooxygenases (COXs), increased endothelium-dependent COXs-mediated contractile responses (EDCF), decreased prostacyclin synthesis, and increased uncoupling of endothelial nitric oxide synthase (eNOS) [12,13,14,15,16]. Thus, COXs seem to contribute to the induction of age-related endothelial dysfunction, in turn leading to increased vascular events. COXs metabolize arachidonic acid (AA) to generate vasoactive prostanoids including prostacyclin and thromboxane A_2_. However, other fatty acids, such as omega-3 polyunsaturated fatty acids (PUFAs) including the two major compounds—eicosapentaenoic acid (EPA) and docosahexaenoic acid (DHA)—are also metabolized by COXs to generate predominantly vasoprotective prostanoids of the three series [17,18]. Previous studies reported that purified EPA, DHA, and their formulations are able to induce potent and sustained endothelium-dependent relaxations in porcine or bovine coronary artery rings by stimulating the formation of nitric oxide (NO) and endothelium-derived hyperpolarization (EDH) [19,20]. Moreover, both the purity and the EPA:DHA ratio are key determinants for the potency of EPA:DHA formulations in inducing endothelium-dependent relaxations in porcine coronary artery rings, with EPA:DHA 6:1 being one of the most potent vasoactive formulations [19]. In addition, in human internal mammary artery rings, EPA:DHA 6:1 but not EPA:DHA 1:1 was able to induce potent relaxations and to inhibit serotonin-induced contractile responses [21]. Similarly, EPA:DHA 6:1 significantly inhibited the platelet-induced serotonin-mediated contractile responses in porcine coronary artery rings [21]. Recently, we have shown that a 2-week intake of EPA:DHA 6:1 significantly improved the established endothelial dysfunction in the mesenteric artery and aorta of aged rats [22].

Therefore, the aim of the present study was to evaluate whether the oral intake of a EPA:DHA 6:1 formulation by middle-aged rats prevents age-related endothelial dysfunction in both the femoral artery and vein, and, if so, to characterize the role of COX1- and COX-2-derived vasoactive prostanoids. The effect of the EPA:DHA 6:1 treatment was also compared to that induced by a different ratio (EPA:DHA 1:1) and to corn oil, an isocaloric control without omega-3 PUFAs.

## 2. Results

### 2.1. EPA:DHA 6:1 Intake Improved Endothelial Relaxation in Femoral Artery and Vein of Middle-Aged-Rats

**Figure 1:**
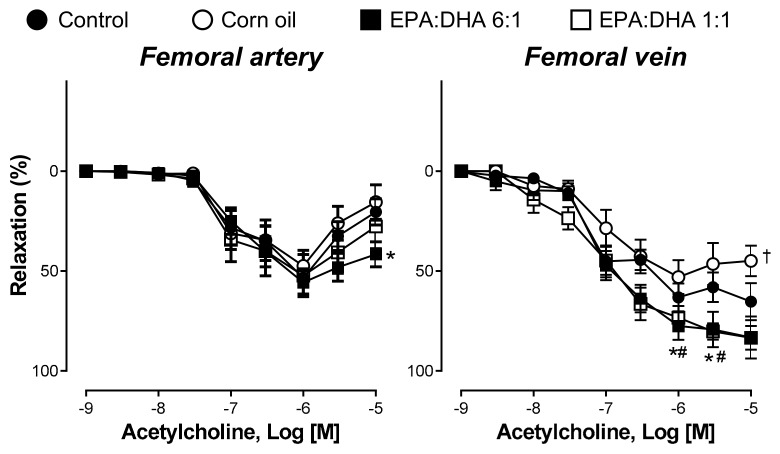
Oral intake of eicosapentaenoic acid (EPA):docosahexaenoic acid (DHA) 6:1 by middle-aged rats improved the endothelial function of the femoral artery and vein. Rings were precontracted with serotonin (5-HT, 1 µM) before the construction of a concentration–relaxation curve to acetylcholine. Results are expressed as percentage of relaxation and given as means ± S.E.M. of ten rats per group. * *p* < 0.05 for EPA:DHA 6:1 vs. control, # *p* < 0.05 for EPA:DHA 1:1 vs. control, and † *p* < 0.05 for corn oil vs. control.

In the femoral artery rings with endothelium of middle-aged control rats, acetylcholine (ACh) caused partial concentration-dependent relaxations, reaching a maximal value of about 51.3% at 1 µM, followed by concentration-dependent contractile responses at concentrations greater than 3 µM (Figure 1). A biphasic relaxation curve to ACh was observed in the corn oil group, the EPA:DHA 1:1 group, and the EPA:DHA 6:1 group (Figure 1). In the corn oil group, the maximal relaxation to ACh (1 µM) amounted to about 47.4%, in the EPA:DHA 1:1 group to about 52.3%, and in the EPA:DHA 6:1 group to about 55.7% (Figure 1). A similar contractile response to ACh at concentrations greater than 3 µM was also observed in the corn oil group and the EPA:DHA 1:1 group, whereas the contractile response was significantly smaller in the EPA:DHA 6:1 group (Figure 1).

In femoral vein rings with endothelium from middle-aged rats, ACh caused concentration-dependent relaxations with a maximal relaxation at 10 µM (Figure 1). In the corn oil group, ACh induced concentration-dependent relaxations up to 1 µM whereas at higher concentrations a contractile response was observed which reached statistical significance at 10 µM (Figure 1). In contrast, the ACh-induced concentration-dependent relaxation was significantly increased in the EPA:DHA 1:1 group and the EPA:DHA 6:1 group compared to that of the control group (Figure 1).

### 2.2. Characterization of Endothelial Dysfunction in Femoral Artery and Vein

**Figure 2:**
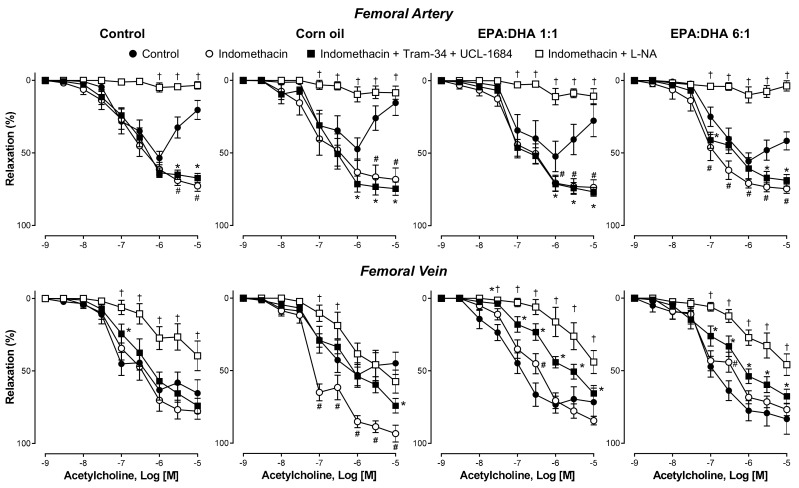
Characterization of the acetylcholine (ACh)-induced endothelium-dependent relaxation of the femoral artery and vein of the control, corn oil, and EPA:DHA 1:1 and EPA:DHA 6:1 groups. Rings were precontracted with serotonin (5-HT, 1 µM) before the construction of a concentration–relaxation curve to ACh. Some rings were incubated for 20 min with indomethacin (10 µM, non-selective inhibitor of cyclooxygenases) in the absence or presence either of TRAM-34 plus UCL-1684 (10 µM each, inhibitors of endothelium-derived hyperpolarization (EDH)-mediated relaxation) or L-NA (300 µM, inhibitor of endothelial nitric oxide synthase (eNOS)). Results are expressed as percentage of relaxation and given as means ± S.E.M. of ten rats per group. # *p* < 0.05 for indomethacin vs. control, * *p* < 0.05 for indomethacin + Tram-34+ UCL-1684 vs. control, and † *p* < 0.05 for indomethacin + L-NA vs. control.

In femoral artery rings, the concentration-dependent relaxations to low concentrations of ACh were not affected by indomethacin (a non-selective cyclooxygenase inhibitor) in the control, corn oil, or EPA:DHA 1:1 groups, whereas those in the EPA:DHA 6:1 group were slightly but significantly increased (Figure 2). In addition, indomethacin abolished the contractile response to higher concentrations of ACh in all four groups, as indicated by greater maximal relaxations reaching about 72.7%, 68.3%, 73.6%, and 74.6% in the control, corn oil, EPA:DHA 1:1, and EPA:DHA 6:1 groups, respectively (Figure 2 and Table S1). The addition of inhibitors of EDH (TRAM-34 plus UCL-1684) to indomethacin-treated femoral artery rings only slightly affected the relaxation to ACh in the four groups (Figure 2). In contrast, the addition of the eNOS inhibitor L-NA to indomethacin-treated rings markedly inhibited the relaxation to ACh in all four groups, indicating a major role of NO (Figure 2).

In femoral vein rings, indomethacin only slightly affected the relaxation to ACh in the control group, the EPA:DHA 1:1 group, and the EPA:DHA 6:1 group, whereas a significant improvement was observed in the corn oil group (Figure 2). The addition of TRAM-34 and UCL-1684 to indomethacin-treated vein rings did not affect the relaxation to ACh in the control group but significantly reduced the relaxations in the corn oil, EPA:DHA 1:1, and EPA:DHA 6:1 groups, suggesting the involvement of EDH to some extent (Figure 2). In contrast, the addition of L-NA to indomethacin-treated vein rings markedly inhibited the ACh-induced relaxation in all groups, indicating a predominant role of NO (Figure 2).

### 2.3. ACh-Induced Relaxation was Improved by COX-1 Inhibition in the Femoral Artery and by COX-2 Inhibition in the Femoral Vein

**Figure 3:**
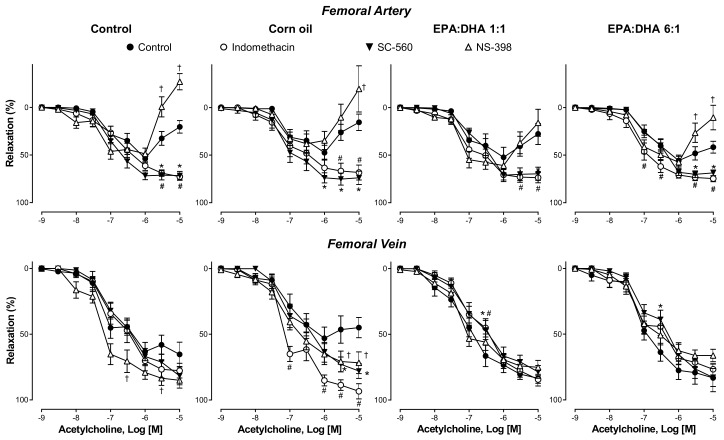
The role of cyclooxygenase (COX)-1 and COX-2 in the ACh-induced endothelium-dependent relaxation of the femoral artery and vein of the control group, the corn oil group, and the EPA:DHA 1:1 and 6:1 groups. Rings were precontracted with 5-HT (1 µM) before the construction of a concentration–relaxation curve to ACh. Some rings were incubated for 20 min with either indomethacin (10 µM, a non-selective inhibitor of cyclooxygenases), SC-560 (0.3 µM, a selective inhibitor of COX-1), or NS-398 (3 µM, a selective inhibitor of COX-2). Results are expressed as percentage of relaxation and given as means ± S.E.M. of ten rats per group. # *p* < 0.05 for indomethacin vs. control, † *p* < 0.05 for NS-398 vs. control, and * *p* < 0.05 for SC-560 vs. control.

Similarly to indomethacin, the ACh-induced relaxation in femoral artery rings was markedly increased by SC-560, a selective COX-1 inhibitor (Figure 3). In contrast, the selective COX-2 inhibitor NS-398 significantly increased the contractile response to the high concentrations of ACh in the control group, the corn oil group, and the EPA:DHA 6:1 group, but not in the EPA:DHA 1:1 group (Figure 3).

In femoral vein rings, the indomethacin-induced improvement of the relaxation to the high concentrations of ACh in the corn oil group was also observed in response to the selective COX-1 and COX-2 inhibitors (Figure 3). The COX-2 inhibitor but not the COX-1 inhibitor slightly but significantly improved the relaxation to ACh in the control group (Figure 3). The COX-1 and COX-2 inhibitors did not affect the relaxation to ACh in the EPA:DHA 1:1 and 6:1 groups (Figure 3).

### 2.4. Sodium Nitroprusside Induced Relaxation in Femoral Artery and Vein

**Figure 4:**
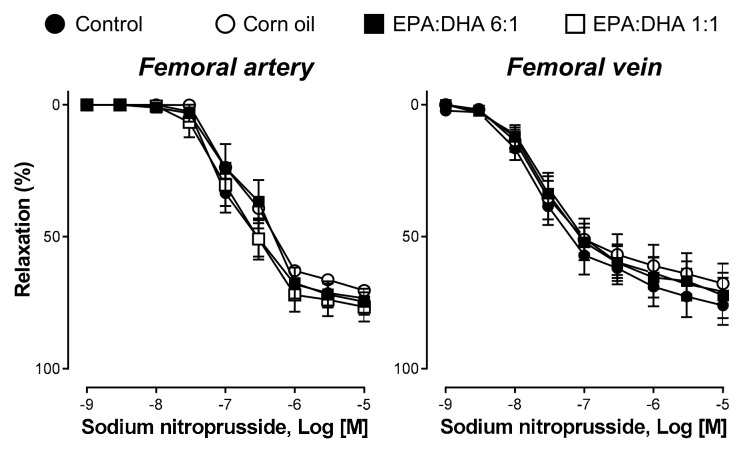
Sodium-nitroprusside-induced concentration-dependent relaxations of the femoral artery and vein of the control group, the corn oil group, and the EPA:DHA 1:1 and 6:1 groups. In the presence of indomethacin (10 µM), TRAM-34 and UCL-1684 (10 µM each), and L-NA (300 µM), rings were precontracted with 5-HT (1 µM) before the construction of a concentration–relaxation curve to sodium nitroprusside, a NO-donor. Results are expressed as percentage of relaxation and given as means ± S.E.M. of ten rats per group.

In the presence of indomethacin, TRAM-34 and UCL-1684, and L-NA, sodium nitroprusside (an NO donor) induced similar concentration-dependent relaxations in femoral artery and vein rings with endothelium in all four groups of rats (Figure 4).

## 3. Discussion

Although increasing age has been associated with the progressive development of endothelial dysfunction in numerous arteries in both preclinical and clinical studies, the impact of ageing on the endothelial function of the vascular bed perfusing the lower limbs such as the femoral artery and vein are scarce. The present findings indicate that in middle-aged rats, endothelial dysfunction was observed in both the femoral artery and vein. In the femoral artery, the endothelial dysfunction was characterized by a reduced maximal relaxation in response to low concentrations of Ach, associated with an increased contractile response at higher ones. A similar endothelial dysfunction has also been observed in the femoral artery of spontaneously hypertensive rats [23]. The results further indicate that the arterial endothelial dysfunction is associated with an endothelial dysfunction in the femoral vein, as indicated by a partial endothelium-dependent relaxation to ACh. The fact that the ACh-induced contractile responses in the femoral artery were abolished by indomethacin indicates the involvement of COX-derived EDCFs. Moreover, the use of selective COX-1 and COX-2 inhibitors indicated a major role of COX-1-derived vasocontractile prostanoids, and that this effect is counteracted by COX-2-derived vasorelaxant prostanoids in the femoral artery. In contrast, in the femoral vein of middle-aged rats, the COX-2 inhibitor but not the COX-1 inhibitor slightly but significantly potentiated the relaxation to ACh, suggesting the involvement of COX-2-derived vasocontracting prostanoids. In addition, the fact that the addition of L-NA to indomethacin-treated rings abolished the relaxation to ACh in the femoral artery and partially reduced that in the femoral vein indicates a predominant role of NO in the femoral artery compared to in the vein.

Moreover, the present study also indicates that a 1-week oral intake of an optimized omega-3 PUFAs formulation containing EPA and DHA at a ratio of 6:1 partially but significantly improved the age-related endothelial dysfunction in both the femoral artery and vein. The beneficial effect of EPA:DHA 6:1 seems to involve a reduced COXs-derived endothelium-dependent contractile response in the femoral artery and an improved endothelium-dependent relaxation in the femoral vein in response to ACh. In addition to the EPA:DHA 6:1 treatment, a similar beneficial effect on the ACh-induced relaxation was also observed with the EPA:DHA 1:1 treatment in the femoral vein but not the femoral artery. In contrast, the isocaloric treatment with corn oil without omega-3 PUFAs resulted in the appearance of slight but significant contractile responses to the highest concentration of ACh in the femoral vein. Altogether, these results indicate that EPA:DHA 6:1 treatment confers not only arterial but also venous protection in the femoral circulation of middle-aged rats, at least in part by modulating the COXs-mediated formation of vasoactive prostanoids.

A previously published study showed that a 2-week intake of EPA:DHA 6:1 improved the endothelial function in the mesenteric artery of old rats with established age-related endothelial dysfunction [22]. Similarly, the intake of EPA:DHA 6:1 prevented the development of endothelial dysfunction in the secondary branch of the mesenteric artery in a rat model of hypertension induced by the chronic infusion of angiotensin II both by reducing the endothelium-dependent COX-mediated contractile response and by improving the EDH- and NO-mediated relaxations [24]. Such a beneficial effect of the EPA:DHA 6:1 treatment on the endothelial function was associated with an increased plasma level of omega-3 PUFAs compared to omega-6 PUFAs, and with a reduction of nearly 65% in the omega-6/omega-3 ratio [24]. The reduction of the omega-6/omega-3 ratio has been shown to exert a beneficial effect on the vascular system by improving the activation of eNOS and by reducing the level of oxidative stress and the inflammatory response [19,25,26]. These effects have been attributed, at least in part, to the fact that EPA and arachidonic acid compete for their metabolism by COXs, with EPA leading to the formation of 3-series prostanoids which are more potent vasodilators (PGI_3_), less pro-aggregating agents (TXA_3_), and have anti-inflammatory properties (PGE_3_) than the 2-series prostanoids derived from arachidonic acid [27]. Moreover, the reduction of the omega-6/omega-3 ratio has also been shown to promote the formation of omega-3-PUFAs-derived anti-inflammatory metabolites such as resolvins and protectins [28,29,30]. In addition to its anti-inflammatory property, resolvin D1 has also been shown to prevent the hyperreactivity induced by endothelin-1 and proinflammatory cytokines such as TNF-α and IL-6 in the human pulmonary artery [31]. In addition, COX-2 mediates the formation of electrophilic fatty acid oxo-derivatives (EFOX) from the omega-3 PUFAs docosahexaenoic acid, docosapentaenoic acid, and docosatetraenoic acid [32]. The fact that the inhibition of COX-2 by NS-398 abolished the generation of EFOXs while the inhibition of COX-1 by aspirin further increased their formation further supports a major role of COX-2-derived EFOX. Both 17-EFOX-D6 and 17-EFOX-D5 derivatives are PPARγ receptor agonists activating the Nrf2-dependent antioxidant response and reducing the inflammatory response [32,33]. Altogether, omega-3 PUFAs might improve endothelial function and promote vascular protection both by reducing the formation of deleterious 2-series prostanoids and by increasing the formation of COXs-mediated omega-3 PUFAs-derived metabolites with vasoprotective and anti-inflammatory properties.

In human clinical trials, supplementation with omega-3 PUFAs has been shown to reduce the risk of cardiovascular death—particularly in patients with the highest cardiovascular risk [34,35,36]. Although several studies did not observe such a beneficial effect (notably a meta-analysis of the Cochrane database), it is important to underline that the studies evaluating the impact of omega-3 PUFAs on the cardiovascular system are very heterogeneous, including patients with different pathologies, both short- and long-term treatment durations, various doses, and non-equivalent quality and purity of the omega-3 PUFAs [37,38]. When converted using a metabolic conversion factor, the dose of 500 mg/kg/day of EPA:DHA 6:1 used in the present study is equivalent to about 5.67 g/day intake by a 70 kg human [39]. This dose is within the range of doses reported in different clinical studies, going from 0.18 to up to 10 g/day [40,41,42,43,44]. In addition, the REDUCE-IT clinical trial has recently reported a decreased risk of cardiovascular events after twice-daily supplementation with 2 g of an EPA ethyl ester (icosapent ethyl) in patients with an established cardiovascular disease, or with diabetes and other risk factors, and on statin therapy with a fasting triglyceride level between 135 and 499 mg/dL [41]. Furthermore, this beneficial effect could be observed across a wide range of baseline triglycerides [45], and was stronger in patients having elevated baseline triglycerides despite moderate-to-high statin therapy intensity [41].

A beneficial effect of omega-3 PUFAs on the peripheral vein reactivity has been observed ex vivo using human saphenous veins, as indicated by the fact that an 18-h treatment of rings with EPA and DHA (100 µM each) reduced their contractile response to noradrenaline in normal veins [46]. In addition, such a pretreatment with EPA and DHA reduced the generation of monocyte chemoattractant protein-1 and tumor necrosis factor-alpha in varicose veins, which are associated with pro-inflammatory conditions [46].

The circulating levels of omega-3 PUFAs seem to negatively correlate with the incidence of VTE. Indeed, the whole-blood omega-3 PUFAs level was shown to predict VTE recurrence and total mortality in elderly patients with acute VTE in two clinical studies based on data from a prospective VTE registry in patients aged 65 and older [47,48]. Furthermore, the risk of recurrent VTE within 6-months follow-up was reduced by more than 60% in patients with higher circulating levels of omega-3 PUFAs, without increased hemorrhagic risk. Conversely, the same group reported in an in vivo study that dietary intake of alpha-linolenic acid was not able to prevent acute venous thrombosis in two mice models of thrombosis, induced by either stenosis of the inferior vena cava or by endothelial lesion in the jugular vein [49]. However, these models used for venous thrombosis are hampered by the fact that they involve severe and irreversible lesions, and hence the endothelial formation of anti-thrombotic compounds may be insufficient to prevent thrombosis. This situation is different from the unprovoked venous thrombosis observed in the human pathology often characterized by no extensive endothelial lesion. Altogether, the present and previous findings suggest that omega-3 PUFAs might exert a potential beneficial effect to prevent venous thrombosis, at least in part by improving the venous endothelial formation of NO and vasoprotective COX-derived prostanoids, and by decreasing that of pro-contractile and pro-thrombogenic prostanoids.

**Figure 5:**
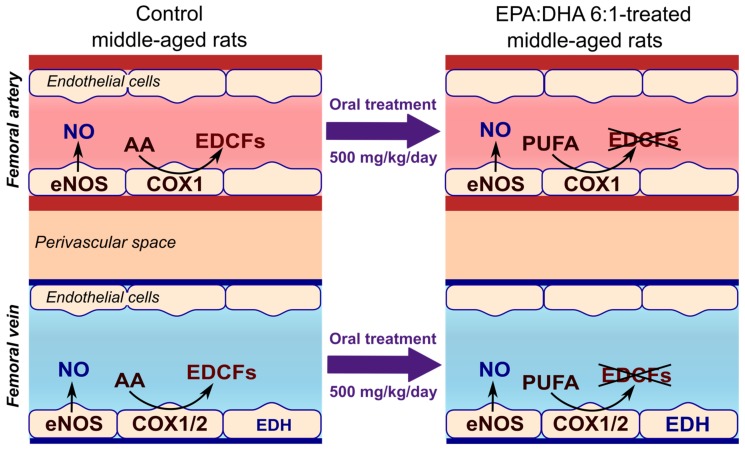
One week oral intake of EPA:DHA 6:1 improved the endothelium function by reducing the COX-derived EDCFs in both femoral artery and vein of middle-aged rats. AA: arachidonic acid; COX: cyclooxygenase; EDCFs: endothelium-derived contractile factors; EDH: endothelium-dependent hyperpolarization; eNOS: endothelial NO synthase; NO: nitric oxide; PUFAs: polyunsaturated fatty acids.

In conclusion, aging is associated with the development of endothelial dysfunction in the artery and vein of the femoral circulation, characterized by reduced endothelium-dependent relaxations and COXs-derived EDCF-mediated contractile responses in middle-aged rats. Oral intake of the omega-3 PUFAs formulation EPA:DHA 6:1 by middle-aged rats for 1 week was able to reduce the COXs-mediated endothelium-dependent contractile response in the femoral artery and improve the relaxation to ACh in both the femoral artery and vein. Taken together, these findings support the concept that EPA:DHA 6:1 might be an interesting new therapeutic approach for the prevention of VTE and its recurrence (Figure 5).

## 4. Materials and Methods

### 4.1. Ethics Statement

This study was performed in accordance with the guidelines on animal care published by the US Institute of Health (Bethesda, MD, USA; NIH publication number 85–23, revised 1996) and the French Legislation. The protocol for this study was approved by the local Ethics Committee (Comité Régional d’Ethique en Matière d’Expérimentation Animale de Strasbourg) and authorized by the French Ministry of Higher Education, Research, and Innovation (authorization #10073-2017053013335510v2).

### 4.2. Preparation of Omega-3 PUFAs Products

Highly purified EPA and DHA products were provided by Pivotal Therapeutics, Inc. (Woodbridge, ON, Canada). EPA:DHA 1:1 and 6:1 (*w/w*) were prepared by mixing the respective purified products in the named ratios. The final solution was aliquoted in amber-colored vials. All the processes were done under nitrogen flux to avoid the oxidation of omega-3 PUFAs.

### 4.3. Chemicals

All chemicals were purchased from Sigma-Aldrich (Sigma Aldrich Chimie S.A.R.L., St.-Quentin-Fallavier, France) except TRAM-34, UCL-1684, NS-398, and SC-560, which were bought from Tocris (Bio-Techne, Abingdon, UK).

### 4.4. Animals and in Vivo Treatment of Rats

Forty male Wistar rats from Janvier labs (Le Genest St Isle, France) were kept in the animal facility and given free access to standard diet and tap water from the age of 12 weeks until they were middle-aged (28 to 32 weeks). They were then randomly divided into four groups of ten rats each and administered daily by gavage 500 mg/kg of either EPA:DHA 6:1, EPA:DHA 1:1, or corn oil (isocaloric control without omega-3 PUFAs, Mazola 100% corn oil) for 7 days. The control group did not received gavage. After 7 days of treatment, the rats were euthanized by an intraperitoneal injection of a lethal dose of ketamine/xylazine (120/40 mg/kg) under isoflurane 2% followed by exsanguination by cardiac puncture before collection of organs.

### 4.5. Vascular Reactivity Studies

To determine the effect of the different treatments on the endothelial function, the reactivity of femoral vein and artery rings was studied using a Halpern–Mulvany myograph (model 620M, Danish Myo Technology A/S, Arhus, Denmark). Briefly, after removal of blood vessels and careful cleaning of connective tissue, calibrated rings (1.8 mm) were cut and suspended between two stainless steel wires with a diameter of 40 µm in an organ chamber filled with oxygenated (95% O_2_, 5% CO_2_) Krebs bicarbonate solution (mM: NaCl 119, KCl 4.7, KH_2_PO_4_ 1.18, MgSO_4_ 1.18, CaCl_2_ 1.25, NaHCO_3_ 25, and D-glucose 11; pH 7.4; 37 °C) for the determination of changes in isometric tension. The femoral vein rings were stretched to 2 mN of tension whereas femoral artery rings were stretched to 3 mN of tension and then allowed to equilibrate for 90 min. After equilibration, the Krebs bicarbonate solution was removed and the rings were immediately exposed to a Krebs bicarbonate solution containing a high concentration of potassium (80 mM) until reproducible contractile responses were obtained. After a 20 min washout period, rings were contracted with serotonin (5-HT, 1 µM) to ~70% of the maximal contraction induced by the high-potassium solution before the addition of acetylcholine (ACh, 1 µM) to test the endothelial function. After washout and a 20 min equilibration period, rings were again contracted with 5-HT before the construction of a concentration–relaxation curve to either ACh or sodium nitroprusside (SNP, an NO donor). In some experiments, rings were exposed to a pharmacological agent for 20 min before contraction with 5-HT. To study the role of cyclooxygenase (COX)-derived vasoactive prostanoids, rings were incubated with indomethacin (10 µM, a non-selective COX inhibitor). To determine the role of COX-1 and COX-2, a selective COX-1 inhibitor (SC-560, 0.3 µM) or a selective COX-2 inhibitor (NS-398, 3 µM) was used. To study NO-mediated relaxation, rings were incubated in the presence of indomethacin (10 µM) and TRAM-34 plus UCL-1684 (10 µM each, inhibitors of IK_Ca_ and SK_Ca_, respectively) to prevent the formation of vasoactive prostanoids and endothelium-derived hyperpolarization (EDH)-mediated relaxation, respectively. The EDH-mediated relaxation was studied in rings incubated with indomethacin and N^G^-nitro-L-arginine (L-NA, 300 µM, an eNOS inhibitor) to prevent the formation of vasoactive prostanoids and NO, respectively.

### 4.6. Statistical Analysis

Values are expressed as means ± S.E.M. Statistical analysis was performed using an analysis of variance followed by the Bonferroni post-hoc test as appropriate using GraphPad Prism (version 5 for Microsoft windows, GraphPad Software, Inc., San Diego, CA, USA). Values of *p* < 0.05 were considered to be statistically significant.

## Supplementary Materials

Supplementary materials can be found at https://www.mdpi.com/1422-0067/21/3/920/s1.

## Abbreviations

VTE	Venous thromboembolism
AA	Arachidonic acid
ACh	Acetylcholine
EPA	Eicosapentaenoic acid
DHA	Docosahexaenoic acid
COX-1	Cyclooxygenase 1
COX-2	Cyclooxygenase 2
EDCF	Endothelium-derived constrictive factors
EDH	Endothelium-derived hyperpolarization
EFOX	electrophilic fatty acid oxo-derivatives
eNOS	Endothelial nitric oxide synthase
PUFAs	Polyunsaturated fatty acids
NO	Nitric oxide
SNP	Sodium nitroprusside

## References

[1] Naess I.A., Christiansen S.C., Romundstad P., Cannegieter S.C., Rosendaal F.R., Hammerstrøm J. (2007). Incidence and mortality of venous thrombosis: a population-based study. J. Thromb. Haemost..

[2] White R.H. (2003). The epidemiology of venous thromboembolism. Circulation.

[3] Heit J.A., Silverstein M.D., Mohr D.N., Petterson T.M., O’Fallon W.M., Melton L.J. (2000). Risk factors for deep vein thrombosis and pulmonary embolism: A population-based case-control study. Arch. Intern. Med..

[4] Gaertner S., Cordeanu E.-M., Mirea C., Frantz A.-S., Auger C., Bilbault P., Ohlmann P., Schini-Kerth V., Stephan D. (2018). Increased risk and severity of unprovoked venous thromboembolism with clustering cardiovascular risk factors for atherosclerosis: Results of the REMOTEV registry. Int. J. Cardiol..

[5] Kearon C., Ageno W., Cannegieter S.C., Cosmi B., Geersing G.-J., Kyrle P.A. (2016). Subcommittees on Control of Anticoagulation, and Predictive and Diagnostic Variables in Thrombotic Disease Categorization of patients as having provoked or unprovoked venous thromboembolism: Guidance from the SSC of ISTH. J. Thromb. Haemost..

[6] Kearon C., Akl E.A., Ornelas J., Blaivas A., Jimenez D., Bounameaux H., Huisman M., King C.S., Morris T.A., Sood N. (2016). Antithrombotic Therapy for VTE Disease: CHEST Guideline and Expert Panel Report. Chest.

[7] Baglin T., Luddington R., Brown K., Baglin C. (2003). Incidence of recurrent venous thromboembolism in relation to clinical and thrombophilic risk factors: Prospective cohort study. Lancet Lond. Engl..

[8] Kyrle P.A., Rosendaal F.R., Eichinger S. (2010). Risk assessment for recurrent venous thrombosis. Lancet Lond. Engl..

[9] Mozaffarian D., Benjamin E.J., Go A.S., Arnett D.K., Blaha M.J., Cushman M., de Ferranti S., Després J.-P., Fullerton H.J., Howard V.J. (2015). Heart disease and stroke statistics—2015 update: A report from the American Heart Association. Circulation.

[10] Hemon F., Fouchard F., Tromeur C., Lacut K., Le Gal G., Mottier D., Couturaud F., Delluc A. (2017). Association between hospitalization for acute medical illness and VTE risk: A lower efficacy of thromboprophylaxis in elderly patients? Results from the EDITH case-control study. Eur. J. Intern. Med..

[11] Tritschler T., Aujesky D. (2017). Venous thromboembolism in the elderly: A narrative review. Thromb. Res..

[12] Matz R.L., de Sotomayor M.A., Schott C., Stoclet J.C., Andriantsitohaina R. (2000). Vascular bed heterogeneity in age-related endothelial dysfunction with respect to NO and eicosanoids. Br. J. Pharmacol..

[13] Tang E.H.C., Vanhoutte P.M. (2008). Gene expression changes of prostanoid synthases in endothelial cells and prostanoid receptors in vascular smooth muscle cells caused by aging and hypertension. Physiol. Genomics.

[14] Tokunaga O., Yamada T., Fan J.L., Watanabe T. (1991). Age-related decline in prostacyclin synthesis by human aortic endothelial cells. Qualitative and quantitative analysis. Am. J. Pathol..

[15] Nakajima M., Hashimoto M., Wang F., Yamanaga K., Nakamura N., Uchida T., Yamanouchi K. (1997). Aging decreases the production of PGI2 in rat aortic endothelial cells. Exp. Gerontol..

[16] Yang Y.-M., Huang A., Kaley G., Sun D. (2009). eNOS uncoupling and endothelial dysfunction in aged vessels. Am. J. Physiol. Heart Circ. Physiol..

[17] Mori T.A. (2017). Marine OMEGA-3 fatty acids in the prevention of cardiovascular disease. Fitoterapia.

[18] Grimminger F., Mayer K., Krämer H.J., Stevens J., Walmrath D., Seeger W. (1993). Differential vasoconstrictor potencies of free fatty acids in the lung vasculature: 2-versus 3-series prostanoid generation. J. Pharmacol. Exp. Ther..

[19] Zgheel F., Alhosin M., Rashid S., Burban M., Auger C., Schini-Kerth V.B. (2014). Redox-sensitive induction of Src/PI3-kinase/Akt and MAPKs pathways activate eNOS in response to EPA:DHA 6:1. PLoS ONE.

[20] Omura M., Kobayashi S., Mizukami Y., Mogami K., Todoroki-Ikeda N., Miyake T., Matsuzaki M. (2001). Eicosapentaenoic acid (EPA) induces Ca(2+)-independent activation and translocation of endothelial nitric oxide synthase and endothelium-dependent vasorelaxation. FEBS Lett..

[21] Zgheel F., Perrier S., Remila L., Houngue U., Mazzucotelli J.-P., Morel O., Auger C., Schini-Kerth V.B. (2019). EPA:DHA 6:1 is a superior omega-3 PUFAs formulation attenuating platelets-induced contractile responses in porcine coronary and human internal mammary artery by targeting the serotonin pathway via an increased endothelial formation of nitric oxide. Eur. J. Pharmacol..

[22] Farooq M.A., Amoura L., Gaertner S., Niazi Z.R., Park S., Qureshi A.W., Oak M.-H., Toti F., Schini-Kerth V.B., Auger C. (2017). The omega-3 EPA:DHA 6:1 formulation improves ageing-related blunted endothelium-dependent relaxations and increased contractile responses in the mesenteric artery: Role of oxidative stress and cyclooxygenases. Biochem. Pharmacol..

[23] Puzserova A., Ilovska V., Balis P., Slezak P., Bernatova I. (2014). Age-related alterations in endothelial function of femoral artery in young SHR and WKY rats. BioMed Res. Int..

[24] Niazi Z.R., Silva G.C., Ribeiro T.P., León-González A.J., Kassem M., Mirajkar A., Alvi A., Abbas M., Zgheel F., Schini-Kerth V.B. (2017). EPA:DHA 6:1 prevents angiotensin II-induced hypertension and endothelial dysfunction in rats: Role of NADPH oxidase- and COX-derived oxidative stress. Hypertens. Res. Off. J. Jpn. Soc. Hypertens..

[25] Simopoulos A.P. (2002). The importance of the ratio of omega-6/omega-3 essential fatty acids. Biomed. Pharmacother. Biomedecine Pharmacother..

[26] Dasilva G., Pazos M., García-Egido E., Gallardo J.M., Rodríguez I., Cela R., Medina I. (2015). Healthy effect of different proportions of marine ω-3 PUFAs EPA and DHA supplementation in Wistar rats: Lipidomic biomarkers of oxidative stress and inflammation. J. Nutr. Biochem..

[27] Wiktorowska-Owczarek A., Berezińska M., Nowak J.Z. (2015). PUFAs: Structures, Metabolism and Functions. Adv. Clin. Exp. Med. Off. Organ Wroclaw Med. Univ..

[28] Seki H., Tani Y., Arita M. (2009). Omega-3 PUFA derived anti-inflammatory lipid mediator resolvin E1. Prostaglandins Other Lipid Mediat..

[29] Hong S., Gronert K., Devchand P.R., Moussignac R.-L., Serhan C.N. (2003). Novel docosatrienes and 17S-resolvins generated from docosahexaenoic acid in murine brain, human blood, and glial cells. Autacoids in anti-inflammation. J. Biol. Chem..

[30] Calder P.C. (2010). Omega-3 fatty acids and inflammatory processes. Nutrients.

[31] Hiram R., Rizcallah E., Sirois C., Sirois M., Morin C., Fortin S., Rousseau E. (2014). Resolvin D1 reverses reactivity and Ca2+ sensitivity induced by ET-1, TNF-α, and IL-6 in the human pulmonary artery. Am. J. Physiol. Heart Circ. Physiol..

[32] Groeger A.L., Cipollina C., Cole M.P., Woodcock S.R., Bonacci G., Rudolph T.K., Rudolph V., Freeman B.A., Schopfer F.J. (2010). Cyclooxygenase-2 generates anti-inflammatory mediators from omega-3 fatty acids. Nat. Chem. Biol..

[33] Malur A., Mccoy A.J., Arce S., Barna B.P., Kavuru M.S., Malur A.G., Thomassen M.J. (2009). Deletion of PPAR gamma in alveolar macrophages is associated with a Th-1 pulmonary inflammatory response. J. Immunol..

[34] Maki K.C., Palacios O.M., Bell M., Toth P.P. (2017). Use of supplemental long-chain omega-3 fatty acids and risk for cardiac death: An updated meta-analysis and review of research gaps. J. Clin. Lipidol..

[35] Lavie C.J., Milani R.V., Mehra M.R., Ventura H.O. (2009). Omega-3 polyunsaturated fatty acids and cardiovascular diseases. J. Am. Coll. Cardiol..

[36] Alexander D.D., Miller P.E., Van Elswyk M.E., Kuratko C.N., Bylsma L.C. (2017). A Meta-Analysis of Randomized Controlled Trials and Prospective Cohort Studies of Eicosapentaenoic and Docosahexaenoic Long-Chain Omega-3 Fatty Acids and Coronary Heart Disease Risk. Mayo Clin. Proc..

[37] Hooper L., Thompson R.L., Harrison R.A., Summerbell C.D., Moore H., Worthington H.V., Durrington P.N., Ness A.R., Capps N.E., Davey Smith G. (2004). Omega 3 fatty acids for prevention and treatment of cardiovascular disease. Cochrane Database Syst. Rev..

[38] Campbell A., Price J., Hiatt W.R. (2013). Omega-3 fatty acids for intermittent claudication. Cochrane Database Syst. Rev..

[39] Reagan-Shaw S., Nihal M., Ahmad N. (2008). Dose translation from animal to human studies revisited. FASEB J..

[40] Delgado-Lista J., Perez-Martinez P., Lopez-Miranda J., Perez-Jimenez F. (2012). Long chain omega-3 fatty acids and cardiovascular disease: A systematic review. Br. J. Nutr..

[41] Bhatt D.L., Steg P.G., Miller M., Brinton E.A., Jacobson T.A., Ketchum S.B., Doyle R.T., Juliano R.A., Jiao L., Granowitz C. (2019). Cardiovascular Risk Reduction with Icosapent Ethyl for Hypertriglyceridemia. N. Engl. J. Med..

[42] Enns J.E., Yeganeh A., Zarychanski R., Abou-Setta A.M., Friesen C., Zahradka P., Taylor C.G. (2014). The impact of omega-3 polyunsaturated fatty acid supplementation on the incidence of cardiovascular events and complications in peripheral arterial disease: A systematic review and meta-analysis. BMC Cardiovasc Disord.

[43] Appel L.J., Miller E.R., Seidler A.J., Whelton P.K. (1993). Does supplementation of diet with “fish oil” reduce blood pressure? A meta-analysis of controlled clinical trials. Arch Intern Med.

[44] Miller P.E., van Elswyk M., Alexander D.D. (2014). Long-chain omega-3 fatty acids eicosapentaenoic acid and docosahexaenoic acid and blood pressure: A meta-analysis of randomized controlled trials. Am. J. Hypertens..

[45] Bhatt D.L., Steg P.G., Miller M., Brinton E.A., Jacobson T.A., Jiao L., Tardif J.-C., Gregson J., Pocock S.J., Ballantyne C.M. (2019). Reduction in First and Total Ischemic Events With Icosapent Ethyl Across Baseline Triglyceride Tertiles. J. Am. Coll. Cardiol..

[46] Daci A., Özen G., Uyar İ., Civelek E., Yildirim F.İ.A., Durman D.K., Teskin Ö., Norel X., Uydeş-Doğan B.S., Topal G. (2017). Omega-3 polyunsaturated fatty acids reduce vascular tone and inflammation in human saphenous vein. Prostaglandins Other Lipid Mediat..

[47] Méan M., Righini M., Jaeger K., Beer H.-J., Frauchiger B., Osterwalder J., Kucher N., Lämmle B., Cornuz J., Angelillo-Scherrer A. (2013). The Swiss cohort of elderly patients with venous thromboembolism (SWITCO65+): Rationale and methodology. J. Thromb. Thrombolysis.

[48] Reiner M.F., Stivala S., Limacher A., Bonetti N.R., Méan M., Egloff M., Rodondi N., Aujesky D., von Schacky C., Lüscher T.F. (2017). Omega-3 fatty acids predict recurrent venous thromboembolism or total mortality in elderly patients with acute venous thromboembolism. J. Thromb. Haemost..

[49] Reiner M.F., Martinod K., Stivala S., Savarese G., Camici G.G., Lüscher T.F., Wagner D.D., Beer J.H. (2015). Dietary omega-3 alpha-linolenic acid does not prevent venous thrombosis in mice. Thromb. Haemost..

