# Supplementary material for: Oral Intake of EPA:DHA 6:1 by Middle-Aged Rats for One Week Improves Age-Related Endothelial Dysfunction in Both the Femoral Artery and Vein: Role of Cyclooxygenases
Source: Int J Mol Sci. 2020 Jan 30;21(3):920. doi: 10.3390/ijms21030920 (PMC7037507; doi:10.3390/ijms21030920)
Supplement: Supplementary file 1 [file ijms-21-00920-s001.pdf]

Table S1: Vasoralaxation values in response to ACh 10^{-6} and 10^{-5} M

	**Control**		**Corn oil**		**EPA:DHA 1:1**		**EPA:DHA 6:1**	
	ACh 10^{-6} M	*ACh 10^{-5} M*	*ACh 10^{-6} M*	*ACh 10^{-5} M*	*ACh 10^{-6} M*	*ACh 10^{-5} M*	*ACh 10^{-6} M*	*ACh 10^{-5} M*
Femoral artery								
Without inhibitor	53,5±12,9	20,4±18,3	47,4±20,4	15,5±22,7	52,3±27,7	27,6±29,1	55,7±16,0	41,6±17,4*
Indomethacin	61,3±13,6	72,7±10,5#	63,3±25,5	68,3±22,0#	70,7±15,3#	73,6±14,9#	70,9±8,8#	74,7±9,0#
Indomethacin + Tram-34 + UCL-1684	63,6±9,6	67,2±9,1#	71,5±15,4#	74,6±12,7#	71,2±15,3#	76,7±8,9#	60,6±20,7	68,8±11,2#
Indomethacin + L-NA	4,8±9,3#	3,3±7,3#	9,64±14,4#	8,48±12,7#	11,0±16,8#	10,7±15,5#	9,9±15,4#	3,68±9,9#
SC-560	71,8±11,7	71,6±12,8#	73,8±14,8#	73,8±19,5#	71,0±18,7	69,6±19,6	68,0±8,8	68,5±7,7#
NS-398	48,6±16,7	-27,1±23,8#	34,5±24,8	-19,6±64,0#	60,9±26,1	15,9±42,2	57,3±16,0	10,4±33,8#
Femoral vein								
Without inhibitor	63,2±18,2	65,3±24,3	53,1±22,2	44,9±20,1*	73,3±15,2*	83,4±15,4	73,5±20,3*	81,0±26,4
Indomethacin	70,2±16,6	77,7±12,3	85,2±9,2#	93,4±12,9#	70,6±13,0	84,4±7,1	68,2±10,8	76,7±10,4
Indomethacin + Tram-34 + UCL-1684	57,0±17,5	74,1±14,6	53,9±17,9	74,2±14,0#	44,1±11,6#	65,6±16,1#	53,8±14,0#	67,6±13,7#
Indomethacin + L-NA	27,3±19,2#	39,6±24,9#	38,4±16,4	57,6±17,7	16,3±22,3#	40,3±19,2#	27,2±11,6#	45,8±16,4#
SC-560	66,9±18,5	81,7±20,3	63,7±19,8	78,0±14,8#	66,4±18,9	78,6±13,7	65,4±23,3	82,9±20,3
NS-398	75,8±15,7	78,1±24,5	65,1±23,7	71,7±24,6#	69,3±10,1	75,1±14,9	62,5±18,0	66,2±14,1

Vasoralaxation data are given as mean ± SD of 5-9 independent experiments. * p<0.05 vs the respective control; # p0<.05 vs. the respective condition without inhibitor.
